# Effect of Volume and Renewal of the Storage Media on the Release of Monomer from Dental Composites

**DOI:** 10.1155/2021/9769947

**Published:** 2021-12-28

**Authors:** Sima Shahabi, Maryam Sayyari, Sima Sadrai, Sara Valizadeh, Hamidreza Hajizamani, Alireza Sadr

**Affiliations:** ^1^Dental Research Center, Dentistry Research Institute, Tehran University of Medical Sciences, Tehran, Iran; ^2^Dental Biomaterials Department, School of Dentistry, Tehran University of Medical Sciences, Tehran, Iran; ^3^Tehran University of Medical Sciences, Tehran, Iran; ^4^Division of Biopharmaceutical and Pharmaceutics and Pharmacokinetics, Department of Pharmaceutics, Faculty of Pharmacy, Tehran University of Medical Sciences, Tehran, Iran; ^5^Dental Research Center, Dentistry Research Institute, Tehran, Iran; ^6^Restorative Dentistry Department, School of Dentistry, Tehran University of Medical Sciences, Tehran, Iran; ^7^Department of Dental Biomaterials, School of Dentistry, Tehran University of Medical Sciences, Tehran, Iran; ^8^Biomimetics Biomaterials Biophotonics & Technology Laboratory, Department of Restorative Dentistry, University of Washington School of Dentistry, Seattle, WA, USA

## Abstract

This study evaluated the effect of the volume and renewing of storage media on monomer leachability from dental composite. Samples of two dental composites (BEAUTIFIL II Gingiva (BG) and Filtek Bulk-Fill Flowable (FBF)) were stored after polymerization in 1 and 3 milt storage media (ethanol/water 75%) for seven days. Refreshing of storage media was done in half of the samples of each group. The amounts of releasing monomers (UDMA, BisGMA, TEGDMA) in storage media were measured by high-performance liquid chromatography (HPLC). Data was analyzed using two-way ANOVA and *t*-test (*α* = 0.05). Elution of TEGDMA and UDMA from both composites was significantly higher in 3 mL storage media. In groups with refreshing of storage media, BisGMA had higher amounts of release. Saturation makes the storage media volume important factor in monomer elution. Refreshing of storage media had significant effect on monomer release before the elution of 50% of total released monomer.

## 1. Introduction

Considering the ever-increasing popularity of composites, there are concerns about the release of composite compounds and their toxicity. In other words, there is a probability that resin materials would release substances such as unpolymerized monomers, additives, and filler component when placed in the oral cavity [1].

In clinical conditions, with a short curing time (usually less than 40 seconds) and the temperature of 37°C, composites will not be fully polymerized, due to the crosslinking reactions that can considerably reduce the movements of the monomers. The degradation process occurring in the oral cavity can also increase the release of certain substances from resin materials, as mechanical, enzymal, and hydraulic processes can break the polymeric chains and release the products of this breakage as monomer or polymer molecules [1, 2].

Resin matrix usually consists of two oligomers: UDMA and BisGMA [3]. BisGMA was synthesized from bisphenol A (BPA) and glycidyl methacrylate (GMA) [4]. The release of monomers and additives can be harmful and might have adverse local or systemic effects. Other than the allergenic properties of monomers, some of the released substances can be cytotoxic, genotoxic, and carcinogenic and might be toxic to the reproduction system [1]. BPA is one kind of endocrine disrupting compound that can cause several health problems [3]. Recognition and evaluation of such risks require knowledge about the exact quantity of the released substances [5].

The vast heterogeneity in the evaluation methods in different studies regarding the measurement of the released compounds from dental composites has had a considerable effect on the results of such studies. While a great number of analytic studies have been carried out, not using the standard means of measurement and varieties in presenting the results prevent an accurate analysis of the quantity of the released monomers [1, 4]. In other words, the amount of monomers released in the oral cavity cannot be precisely determined, rendering it more difficult to evaluate the possible risks of resin composites. The amount of released monomers can differ due to approximately 100,000 factors in different studies.

Although such significant variation can be explained with the fact that different composite materials have been used in each study, many factors play an important role in this matter, such as the storage media (pure ethanol, a mixture of water ethanol, artificial saliva) incubation conditions (temperature and duration) and the methods used for analysis [1, 3, 5]. The most frequently used protocol to measure monomer elution has been described by ISO 10993-12 2010 [6] which suggests that the ratio of the mass of the test sample to the volume of the test storage media should be at least 1 gr: 10 mL [7]. The word “at least” and also the statement of ISO which mentions interference of degradation product with the progress of degradation process should be considered as two major factors in validity of results. However, there is a weak yet significant correlation between the amount of the storage media and the quantity of the released monomers. In other words, the greater the storage media volume is, the higher the monomer concentrations are [1, 5].

This finding can be justified with the saturation of the storage media by the released monomers. Considering the storage media reaching a state of equilibrium and a consequent diminished monomer release, it can be interpreted that the recorded values in the past in vitro studies were speculated with less than the actual values.

Nevertheless, in in vivo conditions, reaching the saturation state does not seem possible, as saliva is continuously being refreshed. One way to rectify this problem in in vitro conditions is to renew the solvent medium within equal intervals, rendering it impossible to reach saturation state [6, 7]. In this study, other than the effect of the volume of storage media on the release of monomers from composites, the effect of renewal of storage media within equal intervals was evaluated. These results are essential for improving experimental protocols regarding monomer release, which will be carried out in future analytic studies with the aim of evaluating the long-term effects of released components from composites in near future. The null hypothesis is that volume and renewing of storage media do not affect monomer leachability.

## 2. Materials and Methods

### 2.1. Preparation of the Specimens

Two types of composites (BEAUTIFUL II Gingiva (BG) and Filtek Bulk-Fill Flowable (FBF)) were selected (Table 1).

64 disk-shaped composite samples (height: 2 mm, width: 6 mm) were prepared in a Teflon mold. Before polymerization, the disks were covered with a glass plate to prevent the formation of an oxygen-inhibited layer. The specimens were cured with the 3 W light curing device high power blue light LED (Guilin Woodpecker, China), with the light output of 500 mw/cm^2^ for 40 seconds according to the manufacturer's instruction.

### 2.2. Storage Media Preparation

The storage media were prepared using pure ethanol (99.99%, Merck, Germany) and water, with the ethanol/water ratio of 75% in two volumes of 1 mL and 3 mL. The composite disks were placed in the storage media according to Table 2 (8 groups and 8 composite discs in each group).

### 2.3. Keeping Samples in Storage Media in Different Methods

**Figure 1:**
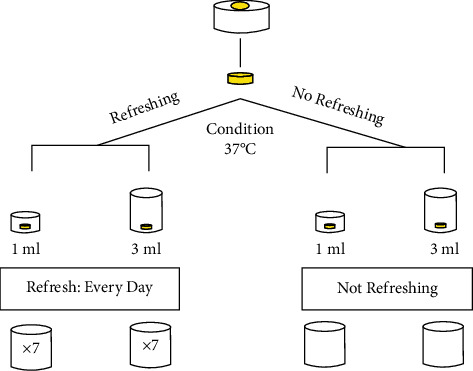
Schematic illustration of the research method used in this study.

The storage media in groups 1, 2, 5, and 6 were incubated for 7 days at 37°C temperature. The storage media in groups 3, 4, 7, and 8 were incubated for 24 hours at 37°C temperature, and, after each 24 hours, all the storage media were taken up for analysis following which the samples were immersed in 1 mL or 3 mL of fresh ethanol/water solution. To be more precise, each composite disc was taken from the solvent, rinsed, and dried by low power air and immersed in fresh solvent. The renewed storage media were again incubated at 37°C temperature. The renewal and incubation of the storage media were repeated every 24 hours for the next 6 days. Schematic illustration of the research method used in this study is available in Figure 1.

### 2.4. Measurement of Monomer Release

**Figure 2:**
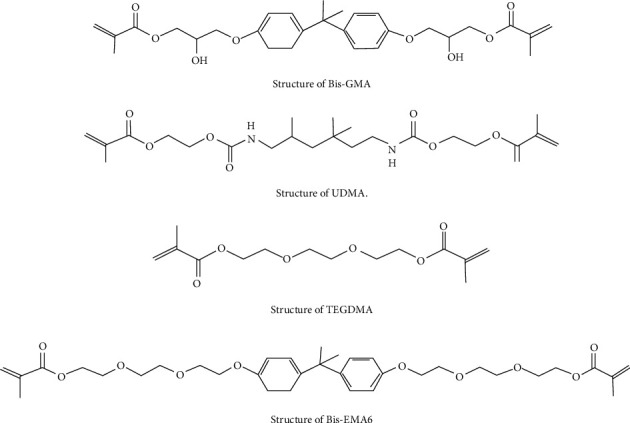
Monomer chemical structure measured in this study.

The amount of released monomer (UDMA, BisGMA, TEGDMA) was evaluated by the HPLC 600E Waters System Controller (Waters, MA, USA) method, through the Perfect Target ODS-3 column (125 mm height, 4 mm width, and silica particle size of 5 *µ*m), with a UV detector at 230 nm wavelength. The mobile phase was 70% acetonitrile and 30% distilled water, at 0.8 mm flow rate, and 20 *µ*lit injection volume at room temperature.  Figure 2 shows the chemical structure of these monomers.

At first, different concentrations of each monomer (0.5–50 *µ*g/lit) were injected into the system, and a standard curve was obtained, which was used to analyze the produced curve of samples. It is noticeable that, in refreshing samples, the sample of each day was injected in the system in order to gain the daily releasing pattern of monomers.

### 2.5. Statistical Analysis

The obtained data were analyzed using the SPSS statistical software 25th version. Continuous variables were presented as mean and standard deviation. Three-way ANOVA and *t*-test were used. The significant level was considered at 0.05.

## 3. Results

Three tracked monomers were detected in all groups of storage media. Volume of solvent did not affect BisGMA release, while TEGDMA and UDMA had significantly higher elution in 3 mL extraction solvent. To be more precise, in samples without refreshing, UDMA from both composites and TEGDMA from FBF eluted in higher amount in 3 mL solvents. To define the effect of composite type in monomer elution, it should be considered that TEGDMA eluted higher from BG composite, while UDMA eluted more from FBF. Refreshing of extraction solvent caused more elution of BisGMA in all samples. Besides, UDMA from FBF in 3 mL eluted more in samples with refreshing in comparison with the samples without refreshing. This result was also true in TEGDMA elution in both solvents' volume from FBF composite. However, for TEGDMA elution from BG in 3 mL solvents, the reverse is true (Table 3).

Daily elution patterns of monomers show that, except UDMA monomer eluted from BG composite in 3 mL solvent, all monomers in all volume had their maximum elution in day one. Meanwhile TEGDMA and UDMA released more than half of their total elution in first day. Composite BG in 1 mL storage media released 83% of its total released TEGDMA in the first day (Tables 4 and 5).

## 4. Discussion

In this study, two different composite materials were used to measure the effect of storage media volume and refreshing of it on monomer elution.

Bulk-fill composites like FBF are in high demand these days because of their increased depth of cure and reduced clinical time of operation [8]. BG can provide esthetics in conservative restorations with gingival recession [9]. Despite the manufacturer's information, elution of TEGDMA from FBF as seen in Pongprueksa et al.'s [10] study and also UDMA from BG were observed. According to Cokic et al. [6], manufacturers do not release the exact component of composites because of trade reasons.

Ethanol was used as storage medium to intensify monomer elution. In aqueous media such as artificial saliva, monomers are released in smaller amounts [11, 12]. Also, FDA declared that ethanol can simulate exposure to nutrition materials such as light drinks and chocolate [13, 14]. According to Van Landuyt et al. [1], UDMA was not detectable during first day of elution.

According to Cokic et al. [6], storage media volume had major effect on monomer elution. The larger the volume of the storage media is, the higher the amounts of monomer elution that happens. Also, incubation time between 7 and 30 days had no significant effect in monomer release. These two observations lead to considering the limitation of monomer solubility in storage media as a major factor in monomer elution. Polydorou et al. [14] measured monomer elution in 1, 7, and 28 days and one year and storage media were refreshed in 28 days. Monomer elution was equal in 28 days and one year which indicates saturation of storage media. In fact, saturation of storage media prevents further elution of monomers. Higher amount of storage media reaches saturation point later, so monomer elution is higher. According to Van Landuyt et al.'s [1] meta-analysis, release of monomers is a chemical equilibrium reaction and in smaller amounts of storage media equilibrium was achieved faster and prevented more monomer release. Constant refreshing of saliva and pulpal fluid in in vivo situation prevent saturation status. High volume of storage media and refreshing of it could be used to overcome this condition.

In this study, 2 volumes of storage media were used to evaluate effect of storage media volume on monomer elution. In order to evaluate the effect of storage media refreshing on monomer elution, in half of the samples, storage media were refreshed daily. Thus, daily pattern of monomer elution was obtained. Alshali et al. [15] measured monomer elution after one day, one month, and three months. In their study, 70% of total three-month elution of TEGDMA and 50% of the total BisGMA elution occurred on the first day. Nalçaci et al. [16] declared that TEGDMA eluted faster than BisGMA in methanol as in one sample group; 92% of TEGDMA eluted in first 6 hours, while at the same time BisGMA reached 57% elution. Sideridou et al. [17] measured monomer elution in 3, 6, and 24 hours and also 3, 6, and 30 days. Similar to the current study, the highest amount of release of TEGDMA and UDMA was observed on the first day, unlike BisGMA.

The first mechanism of monomer elution is elution from the composite surface that occurs in the first 24 hours. Subsequently monomer elution continues with a slower rate, since increasing the volume of polymeric chains and release of unreacted monomers from composite take substantial time [11].

In the current study, TEGDMA and UDMA from FBF and UDMA from BG eluted more in 3 mL storage media (when not refreshing the solvent) because of the faster saturation of 1 mL storage media. In cases of refreshing storage media, two different volumes had no significant difference in reaching saturation status and thus monomer elution.

According to Cokic et al., there are two barriers for monomer release: decreasing the concentration gradient between the sample and the storage media [6] and saturation of storage media with monomers. Thus, rather than the storage media volume and concentration of monomer in the storage media, the concentration of unreacted monomers in the composite plays an important role in reaching equilibrium. Elution of TEGDMA from BG (in cases of not refreshing the solvent) had no significant difference between two volumes of storage media. It can be observed that high concentrations of unreacted TEGDMA in BG led to the continued elution of TEGDMA in 1 mL storage media. Daily pattern of TEGDMA elution also confirms this result. Elution of this monomer from BG, despite of its high rate in elution, continued till day 7. Meanwhile FBF released TEGDMA only on the first day. Elution of BisGMA from FBF and BG was in small amounts, which justifies not reaching saturation in 1 mL volume and not having a significant difference in elution of this monomer in two storage media volumes.

Refreshing of storage media had significant effect on BisGMA elution. Considering that BisGMA could not reach saturation during 7 days because of its small amounts, refreshing of storage media should cause another phenomenon rather than preventing saturation. Refreshing storage media will reduce concentration of monomers in storage media and increase the concentration gradient. BisGMA has a heavy aromatic core, a higher molecular weight [17], and a lower elution rate than TEGDMA and UDMA. BisGMA is the only monomer which eluted lower than 50% of its total elution on the first day. Storage media refreshing and increasing the concentration gradient affect more than 50% of BisGMA's total elution, while for TEGDMA and UDMA this effect is not significant.

TEGDMA elution from FBF is significantly higher in case of refreshing storage media despite its daily elution pattern and rate of elution. Gonzalez-Bonet et al. [18] described that TEGDMA has two hydrolysable ester groups and hydrolyzing these groups creates methacrylate acid (MA), 2-(2-(2-(2-hydroxyethoxy)ethoxy)ethoxy)ethyl methacrylate (TEGMA), and TEG. Finer and Santerre [19] detected TEGDMA products such as TEGMA, triethylene glycol, and methacrylic acid. They suggested that total TEGDMA elution is equal to amount of the main TEGDMA plus its products. It can be obtained that FBF releasing products such as additives and filler components could have hydrolyzed TEGDMA to its products, while refreshing the storage media released TEGDMA and prevented hydrolyzing of this monomer.

3 mL storage media of BG composites at the first day eluted monomers in small amounts, which caused more monomer elution in 1 mL storage media cases with refreshing and also more elution of TEGDMA in 3 mL storage media in cases without refreshing.

## 5. Conclusion

This study was carried out not only to facilitate further studies but also to use them in dental practice. It can be suggested that, in studies related to monomer elution, especially in long-term evaluations, refreshing the storage media should be conducted before first 24 hours, and, after that, due to slower rate of elution, it can be done in longer periods. Higher amount of storage media volume is preferable, while balance between preventing saturation and limit of detection should be considered. Monomers hydrolyzing to their products and measuring their products should be noticed. Moreover, in vitro evaluation of monomer release can be considered as a preface of use of composites in vivo with less harmful effect orally and systemically.

## Figures and Tables

**Table 1:** The properties of the used composites.

Composite brand	Manufacturer	Resin matrix	Filler type	Filler amount
Filtek Bulk-Fill Flowable (1)	3M ESPE GmbH, Germany	BisGMA, BisEMA, Procrylat, UDMA	Zirconia or silica, ytterbium trifluoride	Loading percentage by weight: 64.5%
BEAUTIFIL II Gingiva (2)	Shofu Inc., Japan	BisGMA, TEGDMA	S-PRG filler based on fluoroboroaluminosilicate glass, polymerization initiator, pigments, and others	Loading percentage by weight: 81%

**Table 2:** Test groups.

Group	Composite type	Solvent volume (mL)	Daily renewal
1	Filtek Bulk-Fill Flowable	1	No
2	BEAUTIFIL II Gingiva	1	No
3	Filtek Bulk-Fill Flowable	1	Yes
4	BEAUTIFIL II Gingiva	1	Yes
5	Filtek Bulk-Fill Flowable	3	No
6	BEAUTIFIL II Gingiva	3	No
7	Filtek Bulk-Fill Flowable	3	Yes
8	BEAUTIFIL II Gingiva	3	Yes

**Table 3:** Monomers elution in tested groups (*µ*g).

Monomer		TEGDMA				UDMA				BisGMA			
Volume		1 mL		3 mL		1 mL		3 mL		1 mL		3 mL	
Refreshing		Yes	No	Yes	No	Yes	No	Yes	No	Yes	No	Yes	No
FBF	Mean	0.19	0.01	0.31	0.13	66.99	47.28	76.12	75.39	0.25	0.04	0.18	0.06
	Sd	0.16	0.01	0.07	0.25	8.36	6.92	4.79	25.09	0.45	0.00	0.00	0.01

BG	Mean	0.42	0.36	0.19	0.67	7.83	3.36	6.09	9.64	0.09	0.07	0.19	0.09
	Sd	0.11	0.06	0.05	0.11	1.34	0.65	1.11	12.02	0.01	0.02	0.01	0.01

**Table 4:** Daily release of monomers from FBF (*µ*g).

Monomer		TEGDMA		UDMA		BisGMA	
Volume		1 mL	3 mL	1 mL	3 mL	1 mL	3 mL
Day 1	Mean	0.19	0.31	44.89	47.57	0.03	0.04
	Sd	0.16	0.07	8.73	4.13	0.00	0.00

Day 2	Mean	0.00	0.00	7.60	9.59	0.01	0.03
	Sd	0.00	0.00	0.74	0.18	0.00	0.00

Day 3	Mean	0.00	0.00	5.73	6.31	0.01	0.02
	Sd	0.00	0.00	0.36	0.45	0.00	0.00

Day 4	Mean	0.00	0.00	1.40	2.46	0.01	0.02
	Sd	0.00	0.00	0.03	0.31	0.00	0.00

Day 5	Mean	0.00	0.00	1.29	1.69	0.01	0.02
	Sd	0.00	0.00	0.00	0.51	0.00	0.00

Day 6	Mean	0.00	0.00	3.80	3.39	0.01	0.02
	Sd	0.00	0.00	0.40	0.19	0.00	0.00

Day 7	Mean	0.00	0.00	2.28	4.59	0.01	0.02
	Sd	0.00	0.00	0.82	0.50	0.00	0.00

**Table 5:** Daily release of monomers from BG (µg).

Monomer		TEGDMA		UDMA		BisGMA	
Volume		1 mL	3 mL	1 mL	3 mL	1 mL	3 mL
Day 1	Mean	0.35	0.15	4.85	0.21	0.04	0.05
	Sd	0.11	0.04	1.28	0.56	0.01	0.01

Day 2	Mean	0.04	0.03	0.63	0.97	0.01	0.02
	Sd	0.01	0.01	0.13	0.07	0.00	0.00

Day 3	Mean	0.00	0.00	0.62	0.80	0.01	0.02
	Sd	0.00	0.00	0.15	0.07	0.00	0.00

Day 4	Mean	0.00	0.00	0.00	1.77	0.01	0.02
	Sd	0.00	0.00	0.00	0.56	0.00	0.00

Day 5	Mean	0.00	0.00	0.66	0.69	0.01	0.02
	Sd	0.00	0.00	0.13	0.28	0.00	0.00

Day 6	Mean	0.01	0.01	0.65	0.69	0.01	0.02
	Sd	0.00	0.00	0.08	0.13	0.00	0.00

Day 7	Mean	0.01	0.01	0.42	0.96	0.01	0.02
	Sd	0.00	0.00	0.10	0.13	0.00	0.01

## Data Availability

The data supporting the findings of the article are available from the corresponding author upon request.

## Abbreviations

BG: BEAUTIFIL II Gingiva
FBF: Filtek Bulk-Fill Flowable
BisGMA: Bisphenol A diglycidyl ether dimethacrylate
UDMA: Urethan dimethacrylate
TEGDMA: Triethylene glycol dimethacrylate
HPLC: High-performance liquid chromatography
BPA: Bisphenol A
GMA: Glycidyl methacrylate
LED: Light-emitting diode
ANOVA: Analysis of variance.
